# The Invasive Alien Plant *Solidago canadensis*: Phytochemical Composition, Ecosystem Service Potential, and Application in Bioeconomy

**DOI:** 10.3390/plants13131745

**Published:** 2024-06-24

**Authors:** Danijela Poljuha, Barbara Sladonja, Mirela Uzelac Božac, Ivana Šola, Danijela Damijanić, Tim Weber

**Affiliations:** 1 Institute of Agriculture and Tourism, Karla Huguesa 8, 52440 Poreč, Croatia; barbara@iptpo.hr (B.S.); mirela@iptpo.hr (M.U.B.); danijelad@iptpo.hr (D.D.); 2 Department of Biology, Faculty of Science, University of Zagreb, 10000 Zagreb, Croatia; ivana.sola@biol.pmf.hr; 3 Department of Molecular Life Sciences, University of Zurich, Winterthurerstrasse 190, CH-8057 Zurich, Switzerland; tim.weber2@uzh.ch

**Keywords:** bioactive compounds, bioeconomy, chemical characterisation, ecosystem service, invasive mechanisms, invasive species, phytopharmaceuticals, *Solidago canadensis*

## Abstract

*Solidago canadensis* L. (Canadian goldenrod) is a widely distributed invasive herb from the Asteraceae family. It contains compounds that can change the soil structure and its nutritional components and thus affect indigenous species’ growth, germination, and survival. Consequently, it can pose a major ecological threat to biodiversity. On the other hand, many studies show that this species, due to its chemical properties, can be used for many positive purposes in pharmacy, agriculture, medicine, cosmetic industry, etc. *S. canadensis* contains a diverse array of bioactive compounds that may be responsible for antioxidant, antimicrobial, and anticancer activities. Many studies have discussed the invasiveness of *S. canadensis*, and several chemical and genetic differences between this plant in native and introduced environments have been discovered. Previous ecological and environmental evaluations of the potential of *S. canadensis* as an ecosystem services provider have come out with four promising groups of its products: active extracts, essential oil, fuel, and others. Although identified, there is a need for detailed validation and prioritisation of ecosystem services. This article aims to overview the *S. canadensis* invasive features, emphasising chemical characterisation and its potential for providing ecosystem services. Moreover, it identifies scenarios and proposes a methodology for estimating *S. canadensis* use in bioeconomy.

## 1. Introduction

Invasive alien species (IAS) are not native to a particular ecosystem, and their introduction threatens ecosystems, habitats, or species [1]. By employing ‘competitive traits’, such as wide tolerance to environmental conditions, allelopathy, fast growth, and high fecundity, they permanently change the environment they inhabit [2,3,4], potentially reducing biodiversity and causing local species extinction. IAS can disrupt the delicate balance of ecosystems, leading to significant ecological and environmental changes. However, they can also provide numerous complex services to people [5].

**Figure 1:**
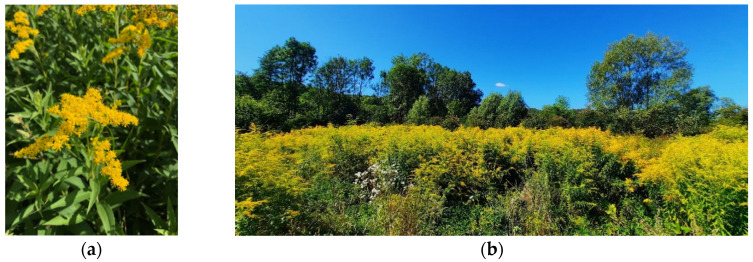
(**a**) *S. canadensis* L. (photo by Mirela Uzelac Božac); (**b**) Widespread occurrence of invasive *S. canadensis* in Istria, Croatia (photo by Danijela Poljuha).

*Solidago canadensis* L. (Canadian goldenrod) (Figure 1a) is an erect rhizomatous perennial plant native to North America [6]. It is one of 138 members of the Genus *Solidago* (Compositae, Asterales) [7], predominantly present in North America, with a few species also natively present in South America, Asia, and Europe. 

**Figure 2:**
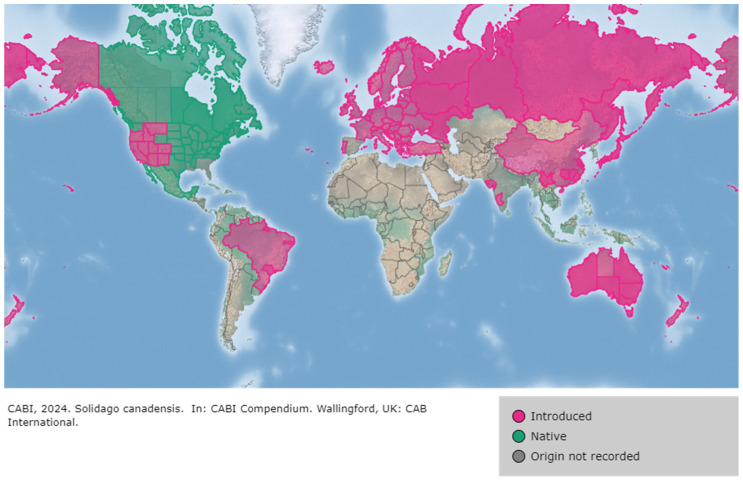
Worldwide distribution of *S. canadensis* (taken from Popay and Parker [11]).

The now-invasive *S. canadensis* was introduced to Europe in 1645 when it was planted in England as an ornamental plant [8]. The species thrives in forest edges, roadsides, and meadows [8,9] (Figure 1b) and is widespread (Figure 2). Since 2004, it has been listed on the EPPO (European and Mediterranean Plant Protection Organization) list of invasive alien plants [10].

For such plants, the EPPO recommends taking control measures and restrictions on sales and raising awareness due to the plant’s high potential to disrupt ecosystems and shift ecosystem services, which are already established [10]. Outside of Europe, China is also facing challenges with *S. canadensis* management, where it has completely dominated some areas and is now recognised as one of the most notorious species in the country [12]. 

Though each invasive alien plant species is unique, ecologists are trying to understand what common mechanisms govern their ability to dominate foreign environments. Canadian goldenrod appears to spread in an invasive manner [13] and could serve as a model invasive species. The case is particularly interesting because it challenges the view of the purely harmful invasive species and forces us to reconsider ecosystems as static networks. The Canadian goldenrod provides several benefits to its new habitats, as studied by Gallardo et al. [9], who looked at the impacts of this species in the French Alps. Authors [9] describe benefits in regulation, provisioning, and supporting ecosystem services by increasing pollinator presence, foraging, and storing carbon. *Solidago* flowers are protandrous and, as such, are an abundant source of nectar and pollen. The flowers thus increase nutrient availability for insects and often extend their foraging seasons [14]. These services are treasured by farmers and beekeepers in the region, observing increased yields in the area [9].

Canadian goldenrod also has noted ‘green synthesis’ capabilities, with noble metal nanoparticles [15,16] and biologically active compounds found in their tissues [17,18]. The plant itself can also be used for the production of natural dyes [19], cellulose [20], biopesticides [21], and as a pharmaceutical source [18,22]. On the other hand, the spread of the species in protected nature reserves threatens endemic and other endangered species [9].

Goldenrods are an example of a species requiring a thorough cost-benefit analysis for effective management. This analysis should consider both the benefits and potential threats of the plant in a specific habitat. Based on this assessment, appropriate actions can be taken to mitigate any negative impacts while maximizing benefits. The outcome brings about the best approximation of a species’ utility in a given area. It serves as a basis for a bioeconomic model that enables us to extract the added value of the plant while minimizing costs and the negative impact of the plant’s presence [23,24].

The present review aims to: (a) provide a general overview of species origin and distribution; (b) give a detailed review of its chemical composition; (c) explain the mechanisms of species invasiveness; (d) provide an overview of the *S. canadensis* ecosystem services; (e) identify scenarios for *S. canadensis* use in bioeconomy; and (f) identify gaps in current knowledge and give recommendations for future research. 

## 2. *Solidago canadensis* Origin and Distribution

*S. canadensis* originated in North America [6], where it is cultivated as an ornamental plant, but is also widely spread as a weed [10]. Virtually ubiquitous in the USA and Canada, *S. canadensis* reaches as high as 65° N in the west of the continent [25,26]. It has been introduced in Europe, Siberia (western), Caucasus, Central Asia, the Himalayas, India, Indochina (Thailand), Malesia, China, Japan, and Australia [26]. According to the latest data, its distribution covers large areas of all continents except Africa and Antarctica (Figure 2) [11]. Apart from *S. canadensis*, there are a few invasive *Solidago* species originating from North America, with *S. gigantea* and *S. altissima* being the most prevalent. However, the taxonomic classification and nomenclature of the *Solidago* genus have undergone numerous changes, influenced by the evolving state of knowledge and varying approaches of different authors. Because of its native range in North America, *S. canadensis* is sometimes treated as an *S. canadensis* complex encompassing several different taxonomic units. The significant morphological variability of these taxa, the formation of hybrids, challenges in distinguishing them, and unresolved taxonomic status all contribute to the complexity of their classification and monitoring [11]. 

Even though *S. canadensis* is one of the most successful plant invaders [27], its further spread is still very likely because it is still available in the web catalogues of commercial nurseries. 

Its dispersal happens in several ways: naturally by seeds and rhizomes, accidentally through human activities, and intentionally for its ornamental value [11]. Within its non-native habitat, *S. canadensis* is observed in various disturbed environments, predominantly along roadsides and railways, urban settings, abandoned fields, and grasslands [11]. It can also be found on the edges of forests, within open forests, along riverbanks, and in habitats disrupted or created by human activity. This species can occur in natural coastal communities, occasionally in semi-natural grassland and spring fen communities [8].

## 3. Phytochemical Composition of *Solidago canadensis*

In recent years, significant attention has been focused on the chemical composition of *Solidago* species, including *S. canadensis*, prompting intensive research efforts [28,29].

In the present review, publicly available literature references on the chemical profile of *S. canadensis* are studied, and sample type and origin, separation methods, and groups of identified compounds are summarised in Table 1. 

Terpenoids constitute a broad and varied category of plant metabolites, encompassing mono-, sesqui-, and diterpenes. These aromatic volatile compounds contain different functional groups, such as alcohols, aldehydes, and ketones. Aromatic and medicinal plants produce secondary metabolites for defence against disease. For example, terpenoids, such as azadirachtin, carvone, menthol, ascaridole, methyl eugenol, toosendanin, and volkensin, have been shown to yield antimicrobial and antifungal properties, as well as insect pest repellent properties [42]. For example, Pandey et al. [42] showed that the oil from the medicinal plant *Chenopodium ambrosioides* has strong antibacterial activity against *Erwinia herbicola* and *Pseudomonas putida* that could be associated with the presence of α-terpinene, p-cymene, and ascaridole. Furthermore, *Lippia alba* essential oil was found to be an effective antifungal agent against *Aspergillus flavus* [43]. Due to their highly lipophilic nature and low molecular weight, terpenoids can disrupt the cell membrane, leading to cell death or inhibiting the sporulation and germination of fungi, on which the antimicrobial activity of essential oils is partly based [44]. Pyrethrins, a small class of specialized metabolites produced in Dalmatian pyrethrum (*Tanacetum cinerariifolium*) are active ingredients of the most widely used botanical insecticides against a wide range of pests [45]. Polyphenols, renowned bioactive compounds, are natural specialized metabolites that constitute an extensive collection of diverse chemicals found in plants, encompassing phytochemicals and enzymes. Moreover, since phenolics are primarily responsible for antioxidant properties and health benefits [46,47], which we pay the most attention to in this review, Table 2 summarises the most abundant individual phenolic compounds in different extracts of *S. canadensis*. 

Extensive scientific research has been conducted on food sources abundant in these compounds, unveiling their potential health benefits [53,54,55,56]. Depending on the type of solvent, *S. canadensis* extract will have a different polyphenols profile [38,39,40]. In the study by Shelepova et al. [40], the proportions of identified polyphenols in extracts of inflorescences and leaves collected in Slovakia are revealed. Even though no statistical analyses are shown, it can be seen that in methanol, ethanol, and acetone extracts, the most predominant polyphenol is rutin (200.45 ± 5.95, 211.20 ± 6.50, and 211.14 ± 5.80 mg/g extract, respectively), while in the water extract, it is chlorogenic acid (834.50 ± 9.75 mg/g extract).

Hydroxycinnamic and hydroxybenzoic acids and flavonoids are the predominant polyphenols in *S. canadensis* herbs (Table 2). If we consider all bibliographic sources included in this paper and listed in Table 2, the total number of so far identified polyphenols is 39 (15 phenolic acids and 24 flavonoids). Among the phenolic acids, hydroxycinnamic acids are prevalent (11), while only four hydroxybenzoic acid compounds are present. In the group of flavonoids, flavonols are predominant (21 different structures), including quercetin and kaempferol and their derivatives.

Analysis of flavonoids in *S. canadensis* flowers revealed the presence of aglycones such as quercetin, kaempferol, and isorhamnetin, with quercetin being the most predominant [40]. Their glycoside forms were also recorded, and quercetin glycosides were dominantly represented both numerically and in concentration. The most noticeable difference between the flower and the leaf was the concentration of the glycosides rutin and hyperoside. Rutin was predominant in flowers and hyperoside was predominant in leaves [57]. Besides the mentioned flavonoids, chlorogenic and caffeic acid were also recorded in the entire aerial parts of *S. canadensis* plants collected in Hungary [38]. The study by Woźniak et al. [39] compared the aerial and underground parts of invasive populations of two equivalent *Solidago* species, *S. canadensis* L. and *S. gigantea* Aiton, in Poland. Quercetin and kaempferol and their glycosides dominated in aerial parts, followed by phenolic acids. The primary constituents in the underground parts identified in crude extracts and medium-polar fractions (ethanol, ethyl acetate, and butanol) were hydroxycinnamic-quinic acid conjugates. Notably, these conjugates predominantly consisted of isomers and derivatives of chlorogenic acid. However, hyperoside, a European Pharmacopoeia standard used for calculating flavonoid content in *Solidaginis herba*, was not recorded in these samples. 

Depending on various factors, including ecological and climatic conditions, the ontogenesis phase, and the specific tissues of the plant, the quantity and chemical profile of essential oils (EOs) can vary significantly. The chemical compositions of EOs extracted from *S. canadensis* leaves, inflorescences, and roots collected in Hungary were compared [30]. The identified compounds were from the group of terpenoids. The EO yield was higher in leaves and inflorescences (0.20% and 0.18%, respectively) than in roots (0.04%). Inflorescences and roots had higher relative percentages of total identified terpenoids (94.3% and 96.2%, respectively) compared with leaves (85.5%), even though statistical analysis was not performed. Monoterpene hydrocarbons predominated based on mass in inflorescences and roots, while oxygenated sesquiterpenes were dominant in leaves. However, when we look at the percentages of the individual identified compounds, the most represented in each of the plant parts were monoterpenes; α-pinene was dominant in inflorescences (29.5 ± 4.5%), limonene in roots (32.7 ± 5.0%), and bornyl acetate in leaves (13.4 ± 2.8%). On the other hand, of the EOs in the green parts of *S. canadensis* collected in Poland, sesquiterpene germacrene D was the most abundant (23.8%) among the 16 annotated compounds. In this sample, two new sesquiterpenes were identified, the major 6-*epi*-β-cubebene and the minor 6-*epi*-α-cubebene [31]. Shelepova et al. [32] screened for the chemical composition of EOs in *S. canadensis* from ten different geographical areas in Eurasia, namely Austria, Ukraine, Kazakhstan, Russia (Moscow Main Botanical Garden, Krasnogorsk district, Penza, Tver, Sakhalin, Altaj and Tula regions). Of all the EOs, mono- and sesquiterpenes were dominant. Specifically, in the aerial parts of nine local *S. canadensis* populations, monoterpene α-pinene was predominant in five (from 24.3% in the Tula region to 52.4% in the Penza region), and bornyl acetate was predominant in one (26.3% in Tver’ region). Sesquiterpene germacrene D was the predominant EO in the other three populations (from 31.8% in Kazakhstan to 36.2% in the Sakhalin region). Plant material was collected at three different developmental stages in the Moscow Main Botanical Garden, and contributed to our understanding of how the chemical profiles of *S. canadensis* EOs evolve at different stages of the plant’s life cycle. The EO content varied from 0.1% in the leaves at the blooming stage, up to 0.70% in the leaves in the vegetative phase, both collected in Moscow Main Botanical Garden. In the blooming phase, inflorescences had more EOs (0.4%) than leaves (0.1%). Regarding the individual components, in the leaves at the vegetative stage, the predominant component was sesquiterpene germacrene D, with a proportion of 39.2%. In comparison, later in the blooming stage, monoterpene bornyl acetate dominated, with a proportion of 29.8%. In the inflorescences, however, the predominant EO was monoterpene α-pinene, with a proportion of 61.2%.

In the research by Amtmann et al. [41], the chemical compound contents in *S. canadensis* flower and honey were compared. GC-MS analyses revealed a higher number of individual aromatic compounds, mostly belonging to the classes terpenes and sesquiterpenes and their derivatives, in honey than in flowers. Germacrene-D was identified as a key compound in both the flowers and the resulting honey produced by bees that forage on *S. canadensis* nectar. Additional volatile compounds in honey were lactones, esters of open-chain acids, linoleic acid, and open-chain and ringed, saturated, and unsaturated hydrocarbons.

## 4. Behind the Invasiveness of *S. canadensis*

In recent years, significant scientific effort has been made to comprehend the mechanisms propelling the spread of invasive plants [58]. The success of many introduced species and the subsequent decline of native ones may be attributed to a combination of various factors [59]. Explaining these mechanisms will not only advance our understanding of plant competition and community ecology but also assist in managing introduced species, fostering conservation, and facilitating the restoration of native communities.

Many plant species are not dominant competitors in their native habitats, but once introduced into new ecosystems, they swiftly become invasive, outcompeting and displacing their new neighbours. A key hypothesis regarding the success of invasive plants suggests that they have evaded the co-evolved natural enemies that typically restrict them in their native regions [60]. Abhilasha et al. [61] conducted a study comparing the populations of *S. canadensis* under similar ecological conditions: in North America, where the species is indigenous, and in Switzerland, where it is invasive. Research showed that despite the lower herbivore pressure in Switzerland, *S. canadensis* had smaller inflorescences, fewer vegetative offspring, and lower growth compared to plants from the native North American range. This suggests that the successful invasion of this species must be based on other morphological traits, such as plant height and prolific production of seeds [62].

Plant population dynamics are strictly connected to climate change, and consequently, they also affect the distribution of invasive species [63]. Cao et al. [57] showed that *S. canadensis* exhibits more plasticity than some native species when it comes to reproductive phenology (prolonging the flowering duration and increasing reproductive investments and root/leaf ratio) in response to simulated climate change. In another research conducted by Bao et al. [64], the primary inquiry revolved around the impact of climate warming and increased precipitation on the growth and tolerance of *S. canadensis* during its establishment phase. In their conclusions, the authors suggested that the increased precipitation may fully counteract the inhibitory impacts of climate warming on the growth of *S. canadensis*, thereby maintaining its high resilience to heat stress. The impact of climate warming on the germination of *S. canadensis* was studied by [65]. The authors concluded that invasive populations of this species were more resistant to rising temperatures or climate change than native populations.

Another theory, also known as the novel weapon hypothesis, suggests that the primary factor driving the establishment and spread of invasive plants within undisturbed communities is the release of novel phytochemicals by the invader [66,67]. The invasive species produce allelopathic compounds with phytotoxic or at least fitness-reducing effects on plant neighbours that have not co-evolved [68]. Moreover, allelopathic effects of *S. canadensis* can be altered by the soil type, plant species identity, the origin of the *Solidago* plants, and the interacting effects of these factors. For example, *S. canadensis* samples from China had greater allelochemical contents than those from the native USA [69]. Recent *S. canadensis* metabolic analysis has revealed the existence of 122 metabolites, including flavonoids, phenylpropanoids, and terpenoids [70], which provide the plant allelopathic potential. Others found that *S. canadensis* affects the growth and seed germination of tested plants (*Lactuca sativa*, *Morus alba*, *Pharbitis nil*, *Brassica campestris*, *Lepidium sativum*, and others) [71,72,73]. The allelopathic effect was also observed in the research by Perera et al. [74] where *S. canadensis* leaf and flower extracts caused lower germination and seedling growth of different grassland plant species such as *Festuca rubra* L., *Phleum pratense* L., *Poa pratensis* L., *Lotus corniculatus* L., *Trifolium repens* L., and *Silene flos-cuculi* (L.) Greuter & Burdet. 

The invasiveness of *S. canadensis* also lies in its ability to alter mineral soil nutrition [28]. Zhang et al. [75] reported that *S. canadensis* invasion decreased nitrate and phosphorus content and reduced soil stability. On the other hand, it increased soil organic carbon, ammonium, NH_4_, and pH. In addition, *S. canadensis* had higher nitrogen uptake capacity than native plants in China, such as *Saccharum officinarum* L., *Lespedeza bicolor* Turcz., and *Sporobolus alterniflora* Loisel [76]. Climate warming combined with nitrogen deposition significantly enhanced the growth performance of *S. canadensis* during the invasion process [77]. In agricultural soils, anthropogenic nitrogen input results in an elevated demand for phosphorus by plants. In such an environment, the competition among plant species may depend on their capacity to effectively utilize soil phosphorus sources [78]. In the case of *S. canadensis*, Wan et al. [78] detected a correlation between increased growth and elevated phosphorus, providing it with a competitive advantage over other plants. Similar findings were reported by Cui et al. [79], where high levels of phosphorus positively impacted the growth of *S. canadensis*. In addition to climate conditions, the nitrogen and phosphorus uptake may depend on the plant’s ploidy. Walczyk et al. [80] investigated diploid, autotetraploid, and autohexaploid varieties of *S. gigantea*, the close relative of *S. canadensis*. The results showed that diploids and polyploids invested more nitrogen and phosphorus into cells, and tetraploids grew more with nitrogen enrichments, suggesting that material costs increased with ploidy level. Within high-pH soils, the roots of *S. canadensis* secrete specialized metabolites that bind with biogenic metal ions and other cations, consequently diminishing nutrient accessibility to neighbouring plants [81,82]. The presence of *S. canadensis* increases the macronutrient cycle rate by boosting aboveground productivity and the accumulation of nutrients in plant biomass. Additionally, it leads to quicker nutrient release from litter and enhances soil microbial activity [83]. The inoculation of *Arbuscular mycorrhizal* fungi with *S. canadensis* increased the growth and its competitive ability to suppress neighbouring native plants [84]. The invasion success of *S. canadensis* may be attributed to its ability to maintain nutrient accumulation in shoots in response to competition with native plant communities [85]. Yang et al. [86] discovered that *S. canadensis* exhibited tolerance to heavy metal stress, while Bielecka et al. [87] found that this species could act as a phytostabilizer of Pb and Zn in heavily contaminated soils. *S. canadensis* also had a higher tolerance to Pb than native plants, which allowed this invasive species to suppress indigenous plant species and could encourage its rapid invasion in Pb-contaminated soil [75]. While various factors contribute to species’ invasiveness, overall, *S. canadensis* is a plant that outgrows native plants in nitrogen and phosphorus-rich soils, with the potential for phytoremediation of heavy metals.

## 5. Ecosystem Services

While changes to the structure and functioning of ecosystems induced by invasion are extensively documented [88], there is a limited understanding of the mechanisms connecting invasive alien species and ecosystem services [89]. The global focus on the repercussions of invasive species on ecosystem services has increased. Ecosystem services refer to natural ecosystems’ benefits to human society or, more broadly, the ecological processes that sustain human life. While the concept of ecosystem services is not new, numerous efforts have been made to quantify and classify these services, especially as new ones have been recognized [90]. Invasive alien species, including *S. canadensis*, can affect natural ecosystems, human habitats, and human well-being positively and negatively. These findings highlight the versatility of *S. canadensis* extracts, positioning them for use in medicine, agriculture, pharmaceutical, and textile industries [91].

### 5.1. Medicinal Ecosystem Services

For centuries, *Solidago* species have been used in European phytotherapy as herbal tea, tincture, or decoct for oral use, as antioxidant, anti-inflammatory, urological, and antiphlogistic remedies [38,92]. Today, the inflorescence of the *Solidago* species is authorized as a product for rheumatic and urinary complaints and for the prevention of cysto- and nephrolithiasis in several European countries [92]. *S. canadensis* is rich in specialized metabolite flavonoids (quercetin, kaempferol, and their glycosides, astragalin, and rutoside), anthocyanidins, triterpene saponins, phenolic acids (caffeic acid, chlorogenic acid, ferulic acid, synaptic, and vanillin acids), and essential oil (cadinene, α and β pinene, myrcene, limonene, sabinene, and germacren D) [92]. Due to their known radical scavenging ability, rutin, quercetin, and chlorogenic acid are particularly important in *Solidago* spp. 

Extracts from aboveground *S. canadensis* parts, including stems, leaves, and inflorescences, demonstrate antibacterial activity against *Listeria monocytogenes*, *Escherichia coli*, *Staphylococcus aureus*, *Salmonella* sp., *Staphylococcus faecalis*, *Bacillus subtilis*, *Klebsiella pneumoniae*, and *Pseudomonas aeruginosa* [93,94]. This observation may contribute to the use of *S. canadensis* extracts as natural antimicrobials in dietary applications [79,80]. Deepa and Ravichandiran [95] found that various extracts of *S. canadensis* exhibited promising antibacterial activity against pathogenic bacteria like *Salmonella typhi*, surpassing the efficacy of ciprofloxacin, a commercial antibiotic, in in vitro disc diffusion assays. Additionally, Mishra et al. [96] investigated the properties of essential oils from *Solidago canadensis* roots using a method similar to the one by Deepa and Ravichandiran [95], revealing significant antibacterial activity against *Streptococcus faecalis* and *Escherichia coli*. 

The research by Šutovská et al. [18] revealed that a polyphenolic–polysaccharide–protein complex isolated from *S. canadensis* could be used as an anti-asthmatic drug. 

The essential oils (EOs) derived from various parts of *S. canadensis*, such as leaves, shoots, and roots, possess valuable features, including antifungal, antimutagenic, antibacterial, and antioxidant activities. These EOs can be used in dietary supplements, cosmetics, nutraceuticals, and pharmaceuticals [24]. The EOs’ chemical compositions include terpenes like germacrene-D, germacrene-A, a-humulene, b-caryophyllene, β-ylangene, 6-epi-α-cubebene, and 6-epi-β-cubebene, as well as α-pinene and limonene [31,36,97,98]. *S. canadensis* EOs also show significant antimicrobial activity, primarily due to their phenolic compounds, flavonoids, terpenoids, and polysaccharides [99].

Research also suggests anticancer potential in *S. canadensis*, with specific compounds, including labdane diterpene, showing cytotoxic activity against human lung cancer A549, colon cancer DLD-1, and normal fibroblasts WS1 cell lines [100].

### 5.2. Agriculture and Food Processing 

Invasive plants may be important foraging resources for honeybees and wild pollinators [101]. As a late-season blooming plant, when many other plants have ceased flowering, *S. canadensis* is a valuable source of nectar for honeybees [102]. *S. canadensis* honey is considered a significant European unifloral honey mostly because of its unique sesquiterpene spectrum and aroma [35]. 

Preparations based on *S. canadensis* are also used in organic agriculture. This species’ essential oils (EOs) have demonstrated efficacy as botanical insecticides [41]. The EOs in *S. canadensis*, with components like α-pinene, germacrene, limonene, and β-pinene, demonstrate antifungal activity against *Botrytis cinerea*, a major contributor to the reduced post-harvest storage life of strawberries. Additionally, they demonstrate antifungal activity against both *Monilinia fructicola* and *Penicillium expansum* compared to Azoxystrobin, a fungicide with broad-spectrum efficacy [21,103]. Notably, the effect of EOs on *B. cinerea* aligned with the observations of [103], who found that the hyphae of *B. cinerea* underwent shrinkage and thinning after treatment with EOs. This mechanism corresponds to the proposed biological mechanism of EO action, leading to fungal cell wall damage and subsequent cell death. Additionally, EOs exhibit promising antibacterial activity against *Pseudomonas fluorescens* and *Clavibacter michiganensis* [21]. *S. canadensis* extracts also demonstrate effectiveness against harmful freshwater cyanobacteria, particularly *Microcystis aeruginosa*, presenting a potential application as an algaecide in small ponds [104]. Diterpenes, also common in *Solidago*, can act as insect antifeedants and growth inhibitors [105]. Given the acceptable ash content level, biochar derived from *S. canadensis* can be used in agriculture for enhancing soil water retention capacity, greenhouse cultivation, composting organic materials, and mitigating unpleasant odours [106]. As a result of torrefaction, one of the main steps when making biochar, the ash content in biochar from the *S. canadensis* increased to 20%.

### 5.3. Fuel 

Biofuels, derived from processing biomass, present a viable alternative renewable energy source compared to fossil fuels [107]. Several articles have considered the use of *S. canadensis* as a methane fuel [108] and pellets [109]. Methane fuel has not shown much potential as a stand-alone product, but it can be combined with cattle slurry [108]. Ciesielczuk et al. [109] concluded that *S. canadensis* pellets could be considered great fuel due to their high calorific value (over 16 MJ kg^−1^), low moisture, low cost, and availability. Similar to those findings, Izydorczyk et al. [110] also confirmed 16.35 MJ/kg calorific value of goldenrod pellets, which is a slightly lower calorific value than the pellets available on the market. Considering the density of goldenrod growth and its calorific value, Ciesielczuk et al. [111] determined that a monoculture spanning one hectare could yield a calorific value of 288.4 GJ. In the research by Wiatrowska et al. [112], *S. canadensis* showed high ethanol efficiency after biomass conversion to bioethanol. *S. canadensis* biomass contains high lignin contents (28.68%), which leads to a high heating value of 19.894 MJ kg^−1^. Recent studies have shown that vegetative and generative parts of *S. canadensis* can be used as raw material in biochar production [106]. The observed results have shown a significant impact of the plant species and the sampled parts on the ash content, volatile matter content, calorific value, and heat of combustion. For biochar produced at a higher temperature, the calorific value and heat of combustion were both higher. 

### 5.4. Others

Besides its application in the energy and agriculture sectors, *S. canadensis* biochar has the potential to be applied in environmental protection [113]. Recent studies indicate that biochar derived from a mixture of stems and leaves of *S. canadensis* has great potential for cadmium adsorption in water treatment. The highest achieved adsorption efficiency of Cd^2+^ was 95.6 ± 0.38% [114].

In the pulp and paper industry, there is an emphasis on replacing commercial fibres with sustainable alternatives. [20] reported using *S. canadensis* (SCL) stems to prepare a cellulose/SCL blend, which increased the stress value by about 7%, increased thermal stability by 75 °C, and reduced strain by about 35% compared to pure cellulose. In addition, *S. canadensis* extracts can be used as natural yellow dyes for textiles since they contain natural yellow dye compounds such as flavonoids quercetin, isoquercitrin, rutin, and kaempferol [19,115,116].

Moreover, this species contains flavonoids like aglycons, glycosides, and acetyl-glycosides, a wide range of polyphenolic compounds, saponins, hydroxycinnamates, and mineral elements, and it is assumed that they can reduce the metal ions to their nanoparticles [19]. Mariychuk et al. [15] reported the use of *S. canadensis* L. leaf extract for the eco-friendly synthesis of triangular and hexagonal gold nanoparticles. Similarly, Botha et al. [16] synthesized Silver (Ag), gold (Au), and Ag–Au bimetallic nanoparticles with the extracts of *S. canadensis* leaves. 

## 6. *Solidago canadensis* as a Resource—Validating Scenarios in Bioeconomy

There have been several attempts to quantify the economic impact of invasive alien species (IAS) at different levels (species, nation, region). The global economic cost of IAS effects is tremendous, having at least quadrupled every decade since 1970 [117]. The impacts of IAS are typically assessed by evaluating environmental harm and financial repercussions. Many positive impacts of IAS on ecosystem services, which are difficult to measure in terms of money, are often disregarded [1]. The impacts of IAS on ecosystem services and sustaining its life support services can be incorporated by joining ecology and economics. Assessing potential solutions for the use of a singular invasive species is a significant challenge, involving the exploration of socio-economic benefits, enhanced added value, and the effective utilization of the species.

Bioeconomy, in a more specialized context, employs economic theories and techniques to enhance the effectiveness of management strategies concerning environmental matters [118]. As the ecosystem services framework of invasive species gains prominence in various aspects of environmental policy decision-making, there is a growing demand for valuation data to support these efforts, encompassing economic and non-economic factors [1].

Invasive species can be treated as local natural resources providing bioproducts with high added value. Bioeconomy has excellent potential and is vital for the sustainable use of resources and the preservation of ecosystems. It is a term that unites ecology, economics, policy, and management of invasive species [23].

**Figure 3:**
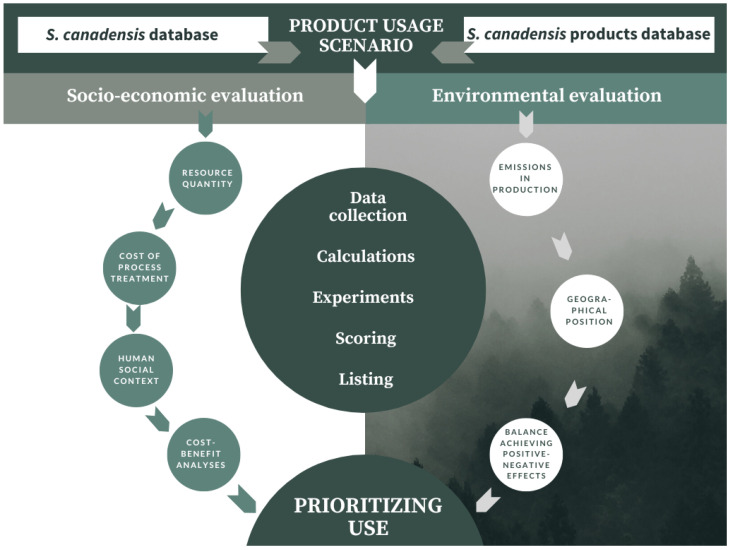
Methodology for Solidago canadensis resource validation (scenarios testing) (adapted from Zihare and Blumberga [91] (Illustration created in Canva).

When a species has multiple bioproducts, a methodology for evaluating the best ones can be helpful. It has been suggested that *S. canadensis* can provide multiple products as ecosystem services, and all parts of this plant can be used to obtain a useful product [24]. Here, we propose a methodology for the evaluation of *S. canadensis* use in bioeconomy (Figure 3). The same methodology can be applied to other invasive plant species or areas. Product evaluation is done considering both environmental and economic aspects.

The evaluation’s objective is to find out if there is a practical possibility of using *S. canadensis* for bioeconomy purposes by identifying the best scenarios with the most significant added value based on the complex data and processing. The best scenario is considered the one with the highest economic benefit and lowest negative impact on the environment or the highest positive impact. The proposed methodology starts with essential information gathering, followed by scenario choosing, socio-economic and environmental evaluation, and decision-making on use type prioritisation. Tested scenarios are extrapolated from the known groups of *S. canadensis* ecosystem services. Zihare and Blumberga [24] grouped all *S. canadensis* products into four groups: extracts, essential oils, fuel, and others. These can be detected as the most promising resources of *S. canadensis*, but inside the last group, “other”, a number of innovative resources can be identified. Further studies are necessary to test and score all feasible scenarios and consequently propose the most promising *S. canadensis* products.

New terms and concepts related to the bioeconomy have been coined in the last decade. While bioeconomy involves the logical and efficient utilization of bioresources, biotechonomy focuses on utilizing bioresources to create new value-added products that meet market demands and are competitive with existing products. Biotechonomy relies on advancing innovative biotechnologies to produce high-value products [119]. Another new concept linked to invasive species parts is the bioeconomic paradox described by Harris et al. [120]. It elaborates that reducing an invasive species’ population will reduce their catch rates and can make their use uneconomical. The authors investigated this seeming paradox through a bioeconomic case study involving the invasive lionfish (*Pterois volitans*).

## 7. Materials and Methods

The choice to focus specifically on *Solidago canadensis*, while setting aside other invasive species of the genus *Solidago* and their hybrids, is driven by our expertise and the prevalence of this species in our region. Our current scientific project investigates the phytochemistry, pharmaceutical potential, and possible new ecosystem services of various invasive species in Istria (Croatia), including *S. canadensis*.

The literature selection criteria for this review were based on the phytochemical composition underlying the mechanisms of *S. canadensis* invasiveness, as well as its potential to provide ecosystem services and its relevance to the bioeconomy. The literature search involved ScienceDirect, Web of Science, and Scopus databases, yielding an initial 2500–3500 entries related to the *Solidago* genus, with 600–900 references specifically pertaining to *S. canadensis*. The search was refined using the following keywords and their derivatives: origin, distribution, invasiveness, phytochemistry, chemical characterization, phenolics, bioactive compounds, ecosystem services, medicine, agriculture, fuel, soil, nanomaterials, energy, and bioeconomy. Three authors independently screened each report, each focusing on one of three areas: phytochemistry, ecosystem services, and bioeconomy. At least two authors reviewed the selection of each entry. Any questionable references and conclusions, as well as the overall approach and findings, were discussed collectively. 

## 8. Conclusions

*Solidago canadensis* L. is a widely distributed invasive plant, whose presence and allelopathic properties can alter soil structure and nutrient composition, affecting the survival of native species, thus posing a significant ecological threat to biodiversity. In this paper, the mechanisms of its invasive properties and differences in native and introduction areas are explained. *S. canadensis* exhibits a wide variety of phytochemicals that play a significant role in both its invasiveness and its potential benefits. This comprehensive overview provides one of the first complete examinations of the chemical compounds present in this invasive species and explores its potential ecosystem services. Special emphasis is placed on its applications in medicine, agriculture, food, and biofuel production, as well as its application in the textile industry and nanotechnology development. The proposed methodology in bioeconomy can be used for prioritising and evaluating *S. canadensis* products as well as for any potential resource of invasive alien plants.

## Figures and Tables

**Table 1:** Sample type and geographical origin of *Solidago canadensis*, identification/quantification methods, phytochemical groups (including the number of identified compounds), with corresponding references.

Sample Type and Origin	Separation Method	Identified Compounds		Reference
		Phytochemical Group	No	
Essential oils from leaves (Hungary)	GC/MS	Terpenoids	66	[30]
Essential oils from inflorescences (Hungary)			71	
Essential oils from roots (Hungary)			69	
Essential oils from the green parts (Poland)	GC-MS and NMR	Terpenoids	16	[31]
Essential oils from leaves at the vegetative stage (Russia, Moscow Main Botanical Garden)	GC-MS		15	
Essential oils from leaves at the blooming stage (Russia, Moscow Main Botanical Garden)			15	
Essential oils from inflorescences (Russia, Moscow Main Botanical Garden)			11	
Essential oils from aerial parts (Moscow region, Krasnogorsk district)			15	
Essential oils from aerial parts (Austria)	GC-MS	Terpenoids	15	[32]
Essential oils from aerial parts (Tver’ region)			16	
Essential oils from aerial parts (Penza region)			14	
Essential oils from aerial parts (Kazakhstan)			15	
Essential oils from aerial parts (Altaj region)			15	
Essential oils from aerial parts (Sakhalin region)			14	
Essential oils from aerial parts (Ukraine)			15	
Essential oils from aerial parts (Tula region)			16	
Essential oils from aerial parts (Slovakia)	GC-MS	Terpenoids	5	[21]
Essential oils from leaves and inflorescences (China)	GC-MS	Terpenoids	6	[33]
Essential oils from the whole plant (China)	GC-MS	Terpenoids	7	[34]
Essential oils from aerial parts (Poland)	GC-MS	Terpenoids	6	[35]
Essential oils from aerial parts (Poland)	GC-MS	Terpenoids	3	[36]
Essential oils from aerial parts (Lithuania)	GC-MS	Terpenoids	6	[37]
Air-dried herbs (Hungary)	HPLC-MS	Flavonoids	7	[38]
		Phenolic acids	2	
Aerial parts (Poland)	UPLC/ESI-MS	Flavonoids	9	[39]
		Phenolic acids	9	
Roots with rhizomes (Poland)		Phenolic acids	9	
Inflorescences and leaves (Slovakia)	LC-MS/MS	Flavonoids	8	[40]
		Phenolic acids	8	
Flowers (Hungary)	GC–MS	Terpenoids	50	[41]
		Compounds with benzene ring	2	
		Open-chain alcohols, aldehydes, and ketones	3	
Honey (Hungary)	GC–MS	Terpenoids	42	
		Compounds with benzene ring	13	
		Open-chain alcohols, aldehydes and ketones	7	
		Lactones	2	
		Esters of open-chain acids	2	
		Fatty acids	1	
		Open-chain and ringed, saturated and unsaturated hydrocarbons	15	

GC-MS = gas chromatography coupled with mass spectrometry, NMR = nuclear magnetic resonance, HPLC-MS = high-performance liquid chromatography coupled with mass spectrometry, UPLC/ESI-MS = ultra-performance liquid chromatography-electrospray tandem mass spectrometry. Values in the brackets indicate the number of identified individual compounds.

**Table 2:** The most abundant phenolic compounds in different extracts of *Solidago canadensis* herb (inflorescences and leaves). Numbers in square brackets indicate the references.

	Polyphenolic Group	Identified Individual Compounds	Presence of Compounds in the Extraction Solvent				
			Methanol	Ethanol	Acetone	Water	Ether: ethanol
Phenolic acids	Hydroxy- benzoic acids 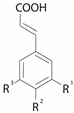	Gallic acid	[40]			[40]	
		Protocatechuic acid	[40]	[40]	[40]	[40]	
		Vanillic acid			[40]	[40]	
		Syringic acid	[40]		[40]		
	Hydroxy- cinnamic acids 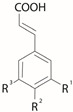						
		5-*O*-Feruloylquinic acid	[39]				
					
					
		Caffeoyl-di-feruloylquinic acid	[39]				
		1-Caffeoylquinic acid	[39]				
		4-Caffeoylquinic acid	[39]				
		5-Caffeoylquinic acid	[39]				
		*t*-Ferulic acid	[40]	[40]	[40]	[40]	
		Chlorogenic acid	[38,39,40,48,49]		[40]	[40]	[40,50]
		Caffeic acid	[38,40,51]		[40]		
		3,4-*O*-Dicaffeoylquinic acid	[39,52]				
		3,5-*O*-Dicaffeoylquinic acid	[39,52]				
		Coumaric acid	[40]	[40]	[40]	[40]	
Flavonoids	Flavonols 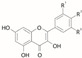	Quercetin	[38,39,40,48,49,51]	[40]	[40]	[40]	
		Quercitrin	[38,39,40,48,49]		[40]	[40]	[40,50]
		Hyperoside	[38,48,49]				
		Isoquercitrin	[38,48,49]			[50]	
		Rutin	[38,39,40,48,49,51]		[40]	[40]	[40,50]
		Quercetin-*O*-hexoside	[39]				
		Quercetin-(acetyl)-hexoside	[48]				
		Quercetin-(rhamnosyl)- hexoside	[48]				
		Quercetin-3-O-(6′-O-acetyl)-β-D-glucopyranoside		[51]			
		Isorhamnetin 3-*O*-hexoside-7-*O*-deoxyhexoside	[39]				
		Isorhamnetin-(acetyl)-hexoside					[50]
		Isorhamnetin-(rhamnosyl)-hexoside	[51]				
					
		Isorhamnetin-3-O-β-D-glucopyranoside		[51]			
		Kaempferol	[39,40,49,51]		[40]	[40]	[40,50]
		Kaempferol-(rhamnosyl)-hexoside isomers	[48]				
		Nicotiflorin	[38]				[50]
		Hesperidin	[40]	[40]	[40]	[40]	
		Afzelin	[38,49]				
		Kaempferol-3-O-(6′-O-acetylyl)- β-D-glucopyranoside		[51]			
		Kaempferol-3-O-β-D-apiofuranoside		[51]			
		Kaempferol-*O*-hexoside-deoxyhexoside	[39]				
	Flavanols 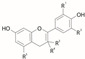	Epicatechin	[40]	[40]	[40]	[40]	
		Catechin	[40]	[40]			
	Flavanones 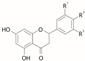	Hydroxy flavanone	[40]	[40]	[40]		

## Data Availability

Not applicable.
